# Topological optimization synergized with a high-activity nano-hydroxyapatite coating to enhance bone regeneration in a porous titanium alloy scaffold

**DOI:** 10.1093/rb/rbag090

**Published:** 2026-05-07

**Authors:** Peng Zhao, Yushuan Jia, Jingming Li, Fengyi You, Kai Zhang, Bo Yuan, Xiangdong Zhu

**Affiliations:** National Engineering Research Center for Biomaterials, Sichuan University, Chengdu 610064, China; College of Biomedical Engineering, Sichuan University, Chengdu 610064, China; National Engineering Research Center for Biomaterials, Sichuan University, Chengdu 610064, China; College of Biomedical Engineering, Sichuan University, Chengdu 610064, China; National Engineering Research Center for Biomaterials, Sichuan University, Chengdu 610064, China; College of Biomedical Engineering, Sichuan University, Chengdu 610064, China; Changzhou Jishuo Medical Device Co., Ltd, Changzhou 213146, China; National Engineering Research Center for Biomaterials, Sichuan University, Chengdu 610064, China; College of Biomedical Engineering, Sichuan University, Chengdu 610064, China; National Engineering Research Center for Biomaterials, Sichuan University, Chengdu 610064, China; College of Biomedical Engineering, Sichuan University, Chengdu 610064, China; National Engineering Research Center for Biomaterials, Sichuan University, Chengdu 610064, China; College of Biomedical Engineering, Sichuan University, Chengdu 610064, China

**Keywords:** porous titanium alloy scaffold, nano-hydroxyapatite, surface modification, osteoinduction, osseointegration

## Abstract

Surface bioinertness-induced fibrosis at and within 3D-printed porous titanium implants critically impairs their repair efficacy. Surface bioactive and evenly distributed coating construction represents an ideal strategy to address this challenge. Herein, a porous titanium alloy scaffold with an appropriate fluid dynamics microenvironment was prepared using computational fluid dynamics simulations and selective laser melting technology. Subsequently, a uniform nano-hydroxyapatite (nHA) coating with about 30 μm thickness and good bonding strength was fabricated on the chemically pretreated scaffold via surface engineering and gradient liquid-phase impregnation. The nHA coating significantly promoted the adhesion, proliferation and osteogenic differentiation of bone marrow mesenchymal stem cells on the scaffold. In a beagle intramuscular model, the rhombic dodecahedron lattice exhibited superior osteoinduction to diamond and hexagonal close-packed lattices, owing to deeper fluid penetration and uniform shear stress. Furthermore, in a rabbit femoral condyle critical-sized defect model, the topology-optimized, surface-modified titanium alloy scaffold exhibited superior interfacial osseointegration and internal bone regeneration. This study not only offers a new approach to addressing current challenges in clinical titanium implant applications but also provides novel insights into the design of 3D-printed functionalized implants.

## Graphical Abstract

**Graphical Abstract:**
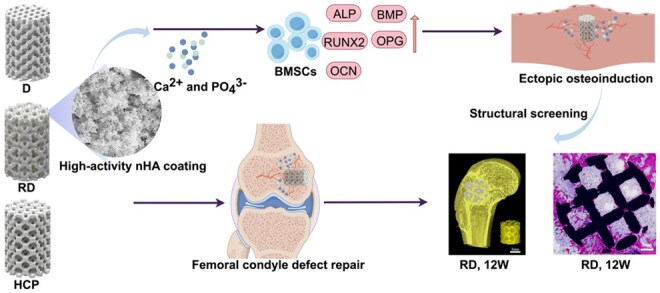

## Introduction

With the accelerating aging of the global population, the incidence of degenerative spinal diseases, joint damage and bone defects caused by trauma or tumor resection continues to rise, leading to an increasingly urgent demand for artificial implants [1–3]. Among various implant materials, titanium and its alloys have become the preferred choice for orthopedic implants due to their excellent strength, corrosion resistance and biocompatibility [4–6]. Nevertheless, due to the significantly higher modulus of titanium-based metals compared to human bone tissue, implantation can trigger a stress-shielding effect, leading to bone resorption and ultimately resulting in the loosening or even failure of the implant [7]. Three-dimensional printing technology enables the fabrication of implants with complex shapes and porous structures, which can reduce the elastic modulus of material to avoid stress shielding while providing space for tissue ingrowth [8, 9]. However, numerous clinical and preclinical studies have shown that the integration strength at the interface of 3D-printed titanium-based implants and internal tissue growth are often limited, still posing risks of implant loosening, infection and failure. The primary cause of this issue is the bioinertness of the titanium metal surface, which lacks the ability to promote or even induce bone tissue regeneration [10, 11].

To address the bioinertness of titanium, researchers have largely employed strategies such as wet chemistry, physical methods or surface coating to modify its surface [12–14]. For example, as early as 2004, Fujibayashi et al. modified porous titanium surfaces using alkali-heat treatment and found that it could enhance *in vitro* bioactivity and *in vivo* osteoinductive capability [15]. However, the improvement was limited, with signs of osteoinduction appearing only after 12 months of ectopic implantation *in vivo*. In response, peptides or other active groups have been grafted onto the titanium surface via chemical grafting to further enhance its bioactivity [16]. Despite delivering significant short-term improvements, these strategies suffer from a lack of lasting effects, frequent incompatibility with subsequent sterilization processes and challenges in clinical application. Additionally, active substances such as copper or cobalt have been incorporated into the titanium matrix using physical methods like plasma implantation. Although these approaches can enhance the osteogenic capabilities of titanium while conferring additional functions such as antibacterial or antitumor properties, the use of localized metal ion strategies may carry potential toxicity risks [17–19].

Compared to chemical or physical surface treatments, constructing bioactive coatings represents a more feasible surface modification strategy. For a long time, titanium-based implants coated with hydroxyapatite (HA) via high-temperature plasma spraying have been widely used in clinical applications and have demonstrated satisfactory repair outcomes [20]. However, plasma spraying is a linear processing technique that is difficult to apply to titanium-based implants with complex surface morphologies or porous structures. Moreover, the extremely high temperatures involved can easily cause phase decomposition of HA, generate thermal stresses leading to coating cracks and produce soluble phases, thereby compromising the long-term stability of the coating [21–24]. Consequently, wet chemical deposition methods such as electrochemical deposition, electrophoretic deposition and sol-gel processes have begun to be explored for constructing bioactive coatings on porous titanium surfaces [25–27]. Nonetheless, these approaches still face limitations such as cumbersome procedures, high costs, poor coating uniformity and low adhesion strength [28]. For example, during electrochemical deposition, the electrolyte parameters are difficult to precisely control, resulting in thin coatings and long processing times [29, 30]. Electrophoretic deposition suffers from insufficient coating uniformity and density and subsequent heat treatment tends to induce microcracks, leading to low bonding strength [31]. The sol-gel method involves lengthy processing cycles and high preparation costs [32]. Therefore, developing a simple process with low cost and suitable bonding strength for preparing hydroxyapatite coatings on porous titanium remains a significant challenge that urgently needs to be addressed.

**Figure 1:**
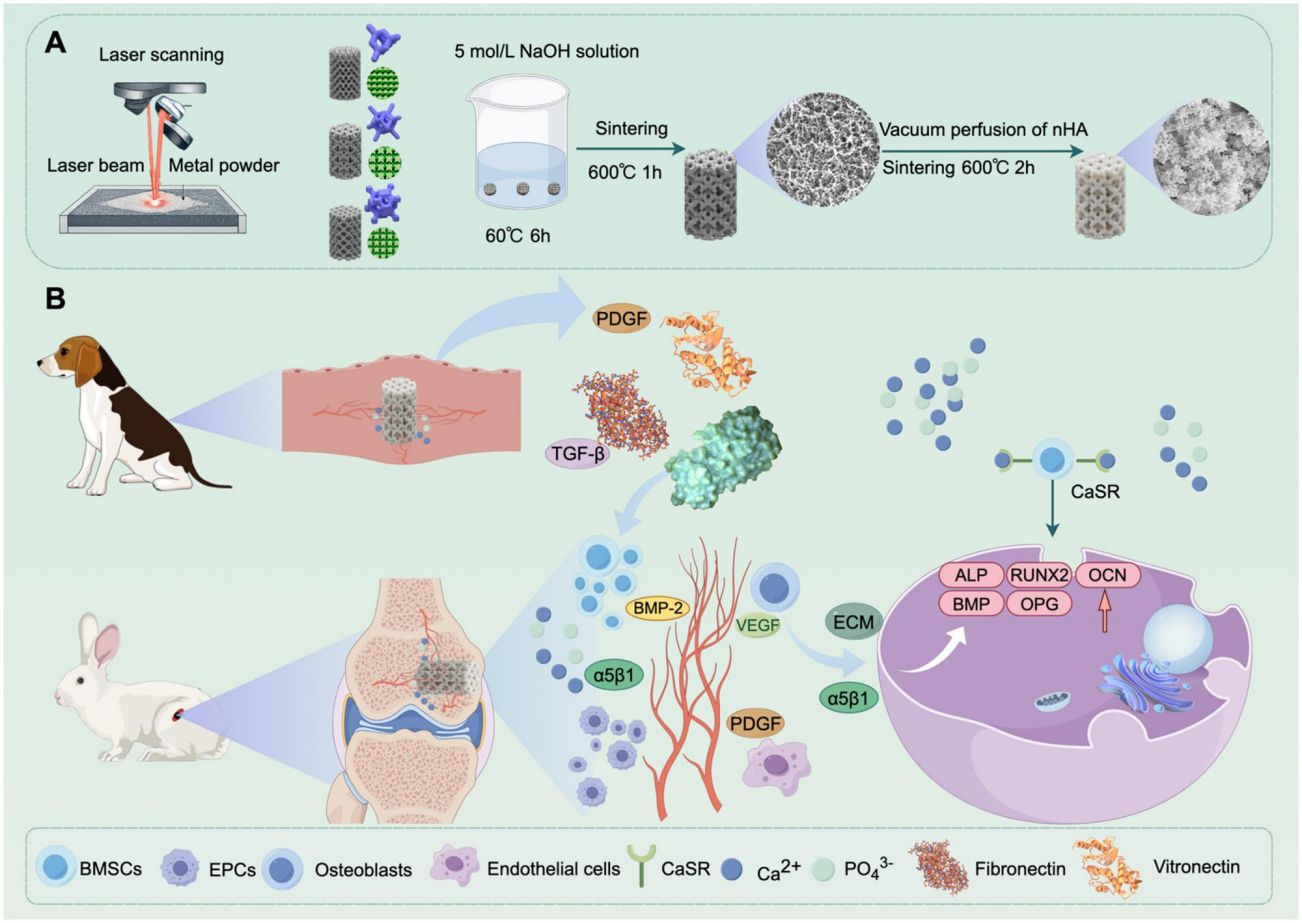
Schematic illustrations of the nHA-coating modification process for porous titanium alloy scaffolds and the subsequent osteogenesis evaluation. (**A**) Fabrication of the porous titanium alloy scaffold and its modification process with the nHA coating. (**B**) Schematic of the ectopic osteoinduction model in the dorsal muscle of beagles and the osseointegration model in femoral condyle defects of New Zealand white rabbits.

Inspired by the tight coating of nano-hydroxyapatite on the bone matrix in natural bone tissue, this study utilizes nHA with unique nanoscale effects, employing a simple slurry casting technique combined with surface engineering strategies to construct a uniform, stable, crack-free and strongly bonded nHA coating on the surface of a 3D-printed porous titanium alloy scaffold. First, based on preliminary research, three lattice structures [diamond (D), rhombic dodecahedron (RD) and hexagonal close-packed (HCP)] were selected and their topological configurations were optimized through CFD simulations. After fabricating the porous titanium scaffold via selective laser melting (SLM), alkali-heat treatment was applied to pretreat the surface, thereby increasing the number of surface contact sites. Subsequently, a gradient vacuum impregnation method was used to uniformly deposit the prepared nHA slurry onto the surface of the porous titanium alloy scaffold. The crystalline morphology of the coating was then regulated through heat treatment. Finally, a beagle canine intramuscular implantation model and a rabbit femoral condyle implantation model were respectively established to evaluate the ability of the 3D-printed porous titanium alloy scaffold to induce bone regeneration and promote osseointegration (Figure 1).

## Materials and methods

### Sample preparation

The titanium alloy samples were fabricated using metal 3D printing (EP-M260, Eplus 3D, China) with powders sized 15–53 µm. According to different topological structures, the as-printed titanium alloy samples can be classified into three types: D-PT, RD-PT and HCP-PT. After support removal, the as-printed samples were sandblasted, ultrasonically cleaned, dried and then subjected to alkali treatment in a 5 mol/L NaOH solution at 60°C for 6 h. Subsequently, the samples were rinsed with deionized water until neutral, followed by ultrasonic cleaning in deionized water and finally dried in a constant-temperature oven at 60°C. The dried samples were sintered in a muffle furnace at 600°C for 1 h and cooled with the furnace to obtain alkali-heat-treated samples (PT-AH).

The nHA coating was fabricated using a vacuum perfusion method. For process optimization and visualization, 3D-printed dense titanium disks were first used, and the optimized protocol was subsequently applied to the porous scaffolds. The perfusion slurry was prepared by uniformly blending a 12 wt.% nHA slurry (Baiameng Bioactive Materials Co., Ltd., China) with a 5 wt.% polyvinyl alcohol (PVA) solution at a volume ratio of 9:1. The alkali-heat-treated samples were placed in a vacuum chamber. Air was evacuated from the chamber to remove gases trapped within the scaffold pores, and the vacuum was maintained (−0.1 MPa). The prepared perfusion slurry was then introduced into the chamber via the pressure differential between the atmosphere and the vacuum, completely submerging the scaffold samples. After 10 min of immersion, the vacuum pump was turned off, and air was reintroduced. Once the pressure equalized, the scaffold samples were transferred to centrifuge tubes and centrifuged to remove excess nHA slurry (300 rpm, 1 min). The samples were then transferred to an oven for drying. This entire perfusion-drying cycle was repeated once to achieve a more uniform coating with moderate thickness. Finally, the coated scaffolds were sintered in a muffle furnace at 600°C for 2 h and cooled with the furnace, yielding the nHA-coated scaffold samples (PT-AH/HA).

In this study, dense disks and porous scaffolds of different sizes were fabricated using the same process according to various testing requirements. Dense disks with dimensions of Φ12 × 2 mm^3^ were used for *in vitro* physicochemical characterization and cell experiments. Porous scaffolds measuring Φ8 × 12 mm^3^ were employed for compression mechanical testing. Porous scaffolds of Φ8 × 4 mm^3^ were utilized for *in vivo* osteoinductivity evaluation, while those sized Φ6 × 6 mm^3^ were applied for *in vivo* osseointegration assessment.

### Material characterization

The surface and cross-sectional microstructures of the samples were observed using a field-emission scanning electron microscope (FE-SEM; S4800, Hitachi, Japan). Elemental composition (O, Ca, P) of the coating surface was qualitatively and semi-quantitatively analyzed via the equipped X-ray energy dispersive spectrometer (EDS), and the calcium-to-phosphorus (Ca/P) ratio was calculated. To evaluate the effect of surface modification on the macroscopic mechanical properties of the 3D-printed porous scaffolds, quasi-static uniaxial compression tests were performed on all scaffold groups using the same universal testing machine (UTM5105X, Sansi Zongheng, China). Testing was conducted until the samples yielded or failed, thereby obtaining their compressive strength and elastic modulus. Three-dimensional topography information and surface roughness parameters of the coating were acquired using an atomic force microscope (AFM; MFP-3D, Asylum Research, USA) on dense titanium alloy disks. The root-mean-square roughness (Rq) was calculated from the scans. The wettability of the samples was evaluated using a water contact angle goniometer (Theta, Biolin, Sweden), with measurements performed in triplicate on three distinct regions for each sample.

Phase composition analysis was conducted using an X-ray diffractometer (XRD; DX-1000, China). Molecular structures and functional groups on the sample surfaces were analyzed by Fourier-transform infrared spectroscopy (FTIR; Nicolet 6700, USA). The bonding strength of the coating was evaluated via a pull-off adhesion test using a polyamide-epoxy modified adhesive (FM1000). Particle size distribution of the original nHA slurry and the degradation products of the nHA coating was measured using a nanoparticle size analyzer (Nano ZS ZEN3600, Malvern Panalytical). The morphology of the original nHA slurry and the degradation products from the scaffold coating were observed using transmission electron microscopy (TEM; JEM-2100Plus, Japan).

### Computational fluid dynamics simulation

To quantitatively evaluate the internal fluid transport characteristics and cell-scale mechanical microenvironment of different porous structures, CFD simulations were performed on scaffolds with the three structures using ANSYS CFX software (2024R1, USA). The fluid domain was a rectangular enclosure containing the complete scaffold, with dimensions of 15 mm × 10 mm × 10 mm. To accurately simulate the fluid environment after scaffold implantation *in vivo*, the material in the fluid domain was set as blood, with a temperature of 37°C, a dynamic viscosity of 0.003 kg/(m·s) and a density of 1060 kg/m^3^. These parameter choices were based on established biofluid mechanics studies to ensure high consistency between the simulation conditions and the actual physiological environment [33]. The boundary conditions were set as follows: a velocity inlet with a flow rate of 1 mm/s and a pressure outlet with a relative pressure of 0 Pa. The Reynolds number (Re) calculated based on these parameters was <10, indicating that the flow state within the scaffold was laminar, which is consistent with the low-velocity percolation characteristics of the bone microenvironment. Permeability was calculated based on Darcy’s law:


(1) k=Q·μ·LA·ΔP,

where k is the permeability, Q is the flow rate, μ is the dynamic viscosity, L is the length, A is the inlet area and ΔP is the pressure drop. Permeability was used to characterize the overall fluid permeability of the structures. The wall shear stress (WSS) values and their distribution contours on all solid walls of the scaffold were extracted to assess the level of mechanical stimulation acting on adhered cells. All calculations were performed with key monitoring parameters stabilized to ensure solution convergence.

### Coating degradation behavior

To simulate the degradation behavior and ion release characteristics of the nHA coating in a physiological environment, samples with a known surface area were immersed in 10 mL of Tris-HCl buffer (pH = 7.4). The containers were sealed with sealing film to prevent solvent evaporation and incubated at 37°C in a constant-temperature incubator for 1, 3 and 7 days, respectively. At each predetermined time point, the extraction solution was collected. The concentrations of calcium (Ca) and phosphorus (P) ions in the solution were determined using inductively coupled plasma mass spectrometry (ICP-MS; Agilent 720, USA) to evaluate the dissolution kinetics and stability of the coating. The immersed samples were dried overnight at 60°C, and then, examined by scanning electron microscopy (SEM) to observe the morphological changes of the coating post-immersion.

### Biocompatibility assessment

Rat BMSCs were isolated from 1-week-old male Sprague–Dawley rats (Chengdu Dashuo Experimental Animal Co., Ltd.). After isoflurane anesthesia, the rats were euthanized by cervical dislocation and disinfected with 75% alcohol. Under aseptic conditions, both femurs and tibiae were harvested, the epiphyses were removed and the bone marrow cavity was flushed with α-MEM medium containing 20% FBS (Gibco, USA) and 1% penicillin/streptomycin (Gibco, USA). The cell suspension was filtered, centrifuged and cultured. Nonadherent cells were removed during the first medium change, and the adherent cells were retained as primary BMSCs. BMSCs were cultured in α-Minimal Essential Medium (α-MEM, Gibco, USA) supplemented with 10% fetal bovine serum (FBS, Gibco, USA) and 1% penicillin/streptomycin (Gibco, USA). The effect of material samples on cell viability was assessed using a CCK-8 assay kit. Under sterile conditions, dry-heat sterilized (200°C, 2 h) samples were placed in a 24-well plate. BMSCs were seeded onto the sample surfaces at a density of 2.0 × 10^4^ cells/well, and each well was supplemented with 1 mL of complete medium. The plate was then transferred to a 37°C cell culture incubator. The culture medium was refreshed every 48 h.

After co-culturing for 1, 3 and 5 days, cytoskeletal staining was conducted with TRITC-conjugated phalloidin (Sigma, USA) and 4',6-diamidino-2-phenylindole (DAPI, Sigma, USA) according to the manufacturer’s protocol. The morphology of BMSCs cultured on different samples was examined by confocal laser scanning microscopy (CLSM; Zeiss LSM880, Germany). Live/dead staining was also carried out using fluorescein diacetate (FDA, Sigma) and propidium iodide (PI, Sigma) and cell viability on various samples was visualized under CLSM. Cell proliferation was evaluated with the Cell Counting Kit-8 (CCK-8, Biosharp, China) and absorbance was read at 450 nm using a microplate reader (BioTek Synergy H1, USA).

### Osteogenic gene expression

Following co-culture of cells with the different material groups for 3 and 7 days, the expression of osteogenic genes, including alkaline phosphatase (ALP), Runt-related transcription factor 2 (RUNX-2), bone morphogenetic protein (BMP), osteocalcin (OCN) and osteoprotegerin (OPG), was quantified using quantitative reverse transcription polymerase chain reaction (qRT-PCR) on a CFX96 Touch system (Bio-Rad, USA). Primer sequences are listed in Supplementary Table S3. Total RNA was first extracted from BMSCs co-cultured with each sample group using the RNeasy Mini Kit (Qiagen, Germany). The extracted RNA was then reverse-transcribed into complementary DNA (cDNA) using the iScript cDNA Synthesis Kit (Bio-Rad, USA). Gene expression was quantified by qRT-PCR using SYBR Green Supermix (TransGen Biotech, China). The relative expression of each target gene was calculated by the comparative ΔΔCt method and normalized to the expression level of the housekeeping gene GAPDH.

### Establishment of the ectopic osteoinduction evaluation model

All animal procedures were strictly conducted in accordance with international ethical guidelines for animal experimentation and were approved by the Institutional Animal Care and Use Committee (IACUC) of the Laboratory Animal Center at Sichuan University (ID: SCU43-2512-05). To evaluate the osteoinductive potential of the nHA-modified porous titanium alloy scaffolds (D-PT-AH/HA, RD-PT-AH/HA, HCP-PT-AH/HA), three male 1-year-old beagle dogs (weighing approximately 12 kg), supplied by Chengdu Dashuo Experimental Animal Co., Ltd. (Chengdu, China), were used for intramuscular implantation in the dorsal region. The as-printed scaffold groups without any modification (D-PT, RD-PT, HCP-PT) served as controls. All implants measured Φ8 × 4 mm^3^. The dogs were anesthetized via an intravenous injection of 3% sodium pentobarbital (30 mg/kg). Under aseptic conditions, five intramuscular pouches were created on each side of the spine in the dorsal region using sterile surgical instruments, resulting in a total of 10 implantation sites per animal. The experimental and control samples of the three structures were implanted accordingly. The muscular and skin incisions were closed layer by layer with sterile surgical sutures, and the wound was disinfected with iodine. To prevent infection, penicillin (40 000 IU/kg) was administered intramuscularly daily for three consecutive days postoperatively. Three months post-implantation, the animals were euthanized via an intravenous overdose of sodium pentobarbital (200 mg/kg). The implant samples and surrounding tissues were harvested for further analysis.

### Establishment of the osseointegration evaluation model

A femoral condyle defect model was established using 35 New Zealand white rabbits (weighing approximately 3 kg), also supplied by Chengdu Dashuo Experimental Animal Co., Ltd. (Chengdu, China), to evaluate the osseointegration performance of the nHA-modified porous titanium alloy scaffolds (D-PT-AH/HA, RD-PT-AH/HA, HCP-PT-AH/HA). In consideration of the 3R principles [34], the rhombic dodecahedron-structured porous titanium scaffolds (RD-PT and RD-PT-AH), which demonstrated the best overall performance in preliminary assessments, were selected as control groups to validate differences in osseointegration among the variously modified samples. All implants measured Φ6 × 6 mm^3^. General anesthesia was induced via an intramuscular injection of Zoletil 50 (0.1 mL/kg) and Domitor (0.05 mL/kg), and maintained with inhaled isoflurane.

The surgical site (bilateral knee joints of the hind limbs) was shaved and disinfected with iodine. A longitudinal incision of approximately 2.5 cm was made on the medial side of the knee. The subcutaneous tissue and muscle were dissected layer by layer to expose the medial femoral condyle. A cylindrical bone defect (6.0 mm in diameter, 6.0 mm in depth) was created perpendicular to the bone surface using a low-speed dental drill under continuous irrigation with sterile saline for cooling. The pre-assigned, sterilized implant was gently press-fitted into the defect cavity, ensuring its top surface was flush with the surrounding subchondral bone plane. The muscle fascia, subcutaneous tissue and skin were sutured sequentially. Postoperative infection prophylaxis was achieved via intramuscular injection of penicillin (40 000 IU/kg) for three consecutive days. At predetermined time points (6 and 12 weeks postoperatively), the animals were euthanized via an intravenous overdose of sodium pentobarbital (200 mg/kg). The distal femurs were harvested bilaterally, and the surrounding soft tissue was carefully removed. The obtained bone-implant complexes were immediately fixed in 4% paraformaldehyde for subsequent analysis.

### μ-CT evaluation and histological observation

The samples in different groups were imaged by a micro-computed tomography system (μ-CT, SCANCO VivaCT80, Switzerland) with a resolution of 14 μm. New bone formation was quantified by bone volume/tissue volume (BV/TV) using the following formula:


(2) $$\frac{BV}{TV}=\frac{V_{b}}{\frac{V_{m}}{1-P}P}\times 100\%,$$

where $V_{b}$ is the volume of new bone tissue within the porous scaffold, $V_{m}$ is the volume of the porous titanium alloy scaffold and P is the porosity of the scaffold (Supplementary Table S1).

Following μ-CT evaluation, the fixed specimens from the osteoinduction study and the femoral condyle implantation study were dehydrated and embedded in polymethylmethacrylate (PMMA). The embedded samples were sectioned into approximately 100 µm thick slices using a diamond saw (SAT001, Aolijin, China). After grinding and polishing, the sections were stained with methylene blue-basic fuchsin. Observation and image acquisition were performed under a fully automated fluorescence scanning microscope (Austar43, Aimite, Chengdu, China). Histological evaluation of the obtained microscope images was conducted using Image-Pro Plus software (Media Cybernetics, USA) for quantitative analysis of new bone formation. The success rate of ectopic osteoinduction was also statistically analyzed for each scaffold group.

Both the osteoinductive area and the bone ingrowth area were calculated using formula (3): the area of newly formed bone (A) within the voids of the scaffold in a single slice was measured and compared to the total area of all pore structures (B) inside the scaffold.


(3) AB×100%.

The bone-implant contact (BIC) rate was calculated using formula (4): the length (l) of direct contact between the newly formed bone tissue and the outer contour of the scaffold in a single slice was measured and compared to the total perimeter (L) of the scaffold’s outer contour.


(4) lL×100%.

### Statistical analysis

Unless otherwise specified, all data are presented as the mean ± standard deviation (SD) of three independent experiments. Statistical analyses were performed using SPSS software (version 16.0). Comparisons between groups were conducted by one-way analysis of variance (ANOVA) followed by Tukey’s *post hoc* test. Differences were considered statistically significant when the *P *< 0.05.

## Results

### Preparation and characterization of 3D-printing titanium alloy scaffolds

Due to their respective structural benefits, the D, RD and HCP structures are commonly used in bone repair scaffold design, with the D structure promoting pore interconnectivity and permeability, the RD structure enhancing structural efficiency and mechanical properties and the HCP structure mitigating stress concentration through its high nodal density [35–37]. Therefore, based on these three structures, we explored different structural combinations by varying parameters such as unit cell size and strut diameter. The fabricated scaffolds were subjected to compressive strength testing according to the designs, ultimately leading to the selection of structural combinations with superior mechanical performance (Supplementary Table S1).

**Figure 2:**
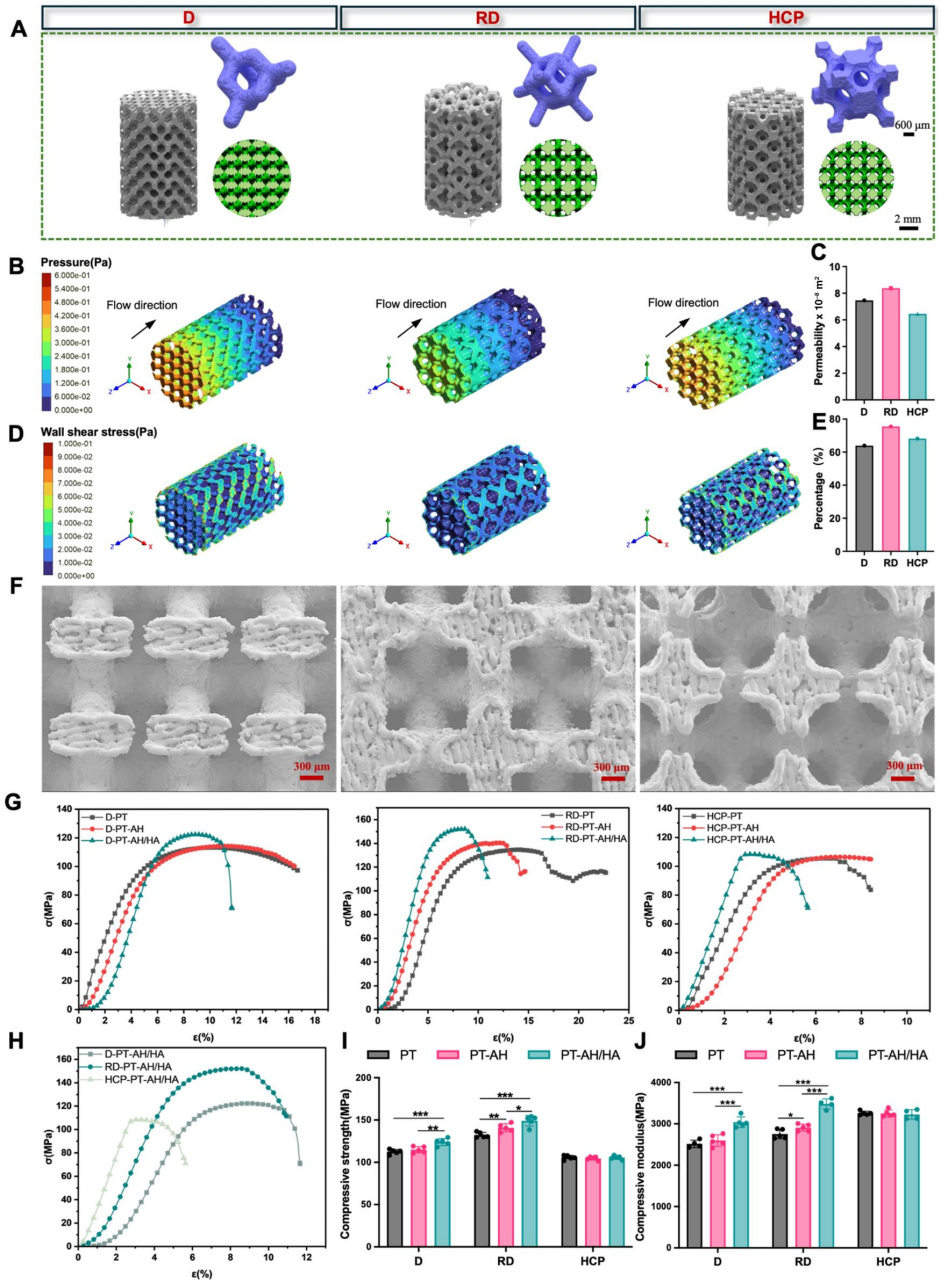
Structural characteristics, surface modification and mechanical performance of the porous titanium alloy scaffolds. (**A**) Schematics showing scaffold design, unit cell and cross-section. (**B**) Pressure distribution from CFD simulation. (**C**) Simulated permeability. (**D**) Wall shear stress (WSS) distribution. (**E**) Percentage of scaffold surface area with WSS < 10 mPa. (**F**) SEM images of surface morphology. (**G**) Stress–strain curves of the scaffolds. (**H**) Comparative stress-strain curves of the three scaffolds after nHA modification. (**I**, **J**) Compressive strength and modulus after different modification steps. (*P < 0.05, **P < 0.01, ***P < 0.001).

The design models, unit cells and cross-sectional profiles of the three structures clearly demonstrate their distinct spatial configurations (Figure 2A). To optimize and quantify their fluid permeability and mechanical microenvironment, computational fluid dynamics (CFD) simulations were performed. The simulated pressure distribution (Figure 2B) reveals a gradual pressure decrease along the flow direction. Under identical inlet flow velocity, the RD structure presented the smoothest flow path with the smallest pressure drop, whereas more pronounced internal pressure gradients were observed in the D and HCP structures. This trend was fully corroborated by the calculated Darcy permeability (Figure 2C), with the RD structure displaying the highest value of 8.38 × 10^−8^ m^2^. The wall shear stress (WSS) distribution (Figure 2D) indicates a relatively uniform WSS distribution for the RD structure, whereas the D and HCP structures showed localized areas of high WSS concentration at narrow constrictions. Further statistical analysis of the proportion of the surface area experiencing WSS below 10 mPa (low-stress region) revealed that the RD structure had the highest percentage of this low-stress region, reaching 75.51% (Figure 2E).

The as-fabricated surfaces of all three scaffolds displayed characteristic SLM morphology, with partially melted titanium particles and micro‑porosity evident in SEM images (Figure 2F). This inherent roughness provided a favorable substrate for subsequent nHA deposition and anchoring. Quasi‑static uniaxial compression tests were performed on pristine, alkali‑heat treated and nHA‑coated scaffolds of each structure (*n* = 5 per group) to assess compressive properties and elastic modulus. Stress–strain curves (Figure 2G and H) revealed that the RD scaffolds possessed superior compressive strength and toughness relative to the D and HCP designs. As shown in Figure 2I, the compressive strength of D‑PT‑AH/HA and RD‑PT‑AH/HA groups exceeded that of their alkali‑treated and pristine counterparts. Among the three architectures, the RD groups consistently achieved the highest compressive strength across all surface conditions, with RD‑PT‑AH/HA reaching 149.16 ± 6.27 MPa, a value greater than that of the corresponding modified groups of the other two structures. In contrast, the compressive strength of HCP scaffolds did not differ significantly among treatment groups. Notably, the compressive modulus values for all scaffold groups fell within the approximate range of 2.5–3.5 GPa (Figure 2J).

**Figure 3:**
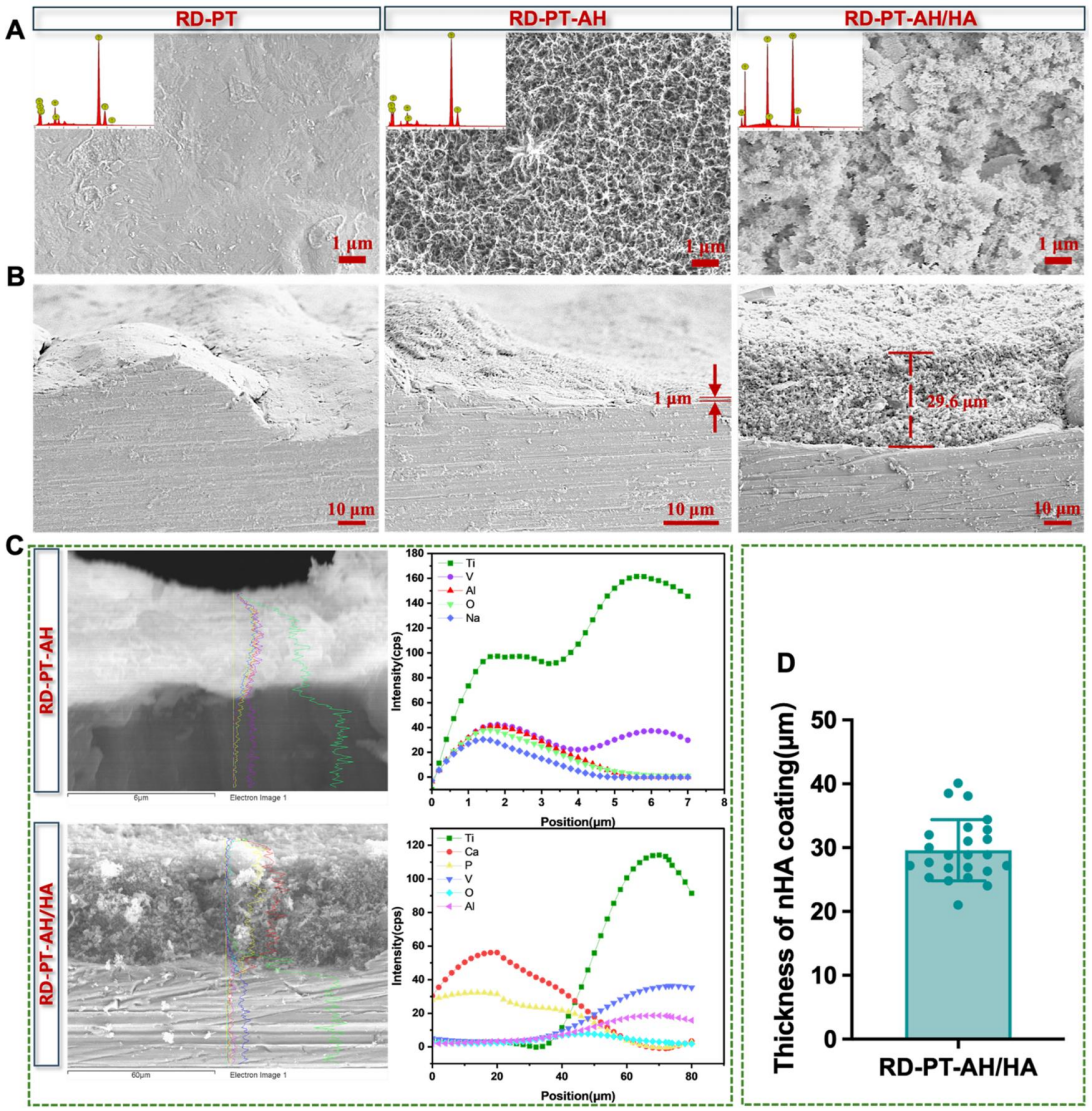
Morphology and elemental variation on the surface and cross-section of the samples. (**A**) Surface morphology of the scaffold. (**B**) Cross-sectional morphology and coating thickness. (**C**) Elemental variation across the sample cross-section obtained by EDS line scanning. (**D**) Numerical distribution of nHA coating thickness.

**Figure 4:**
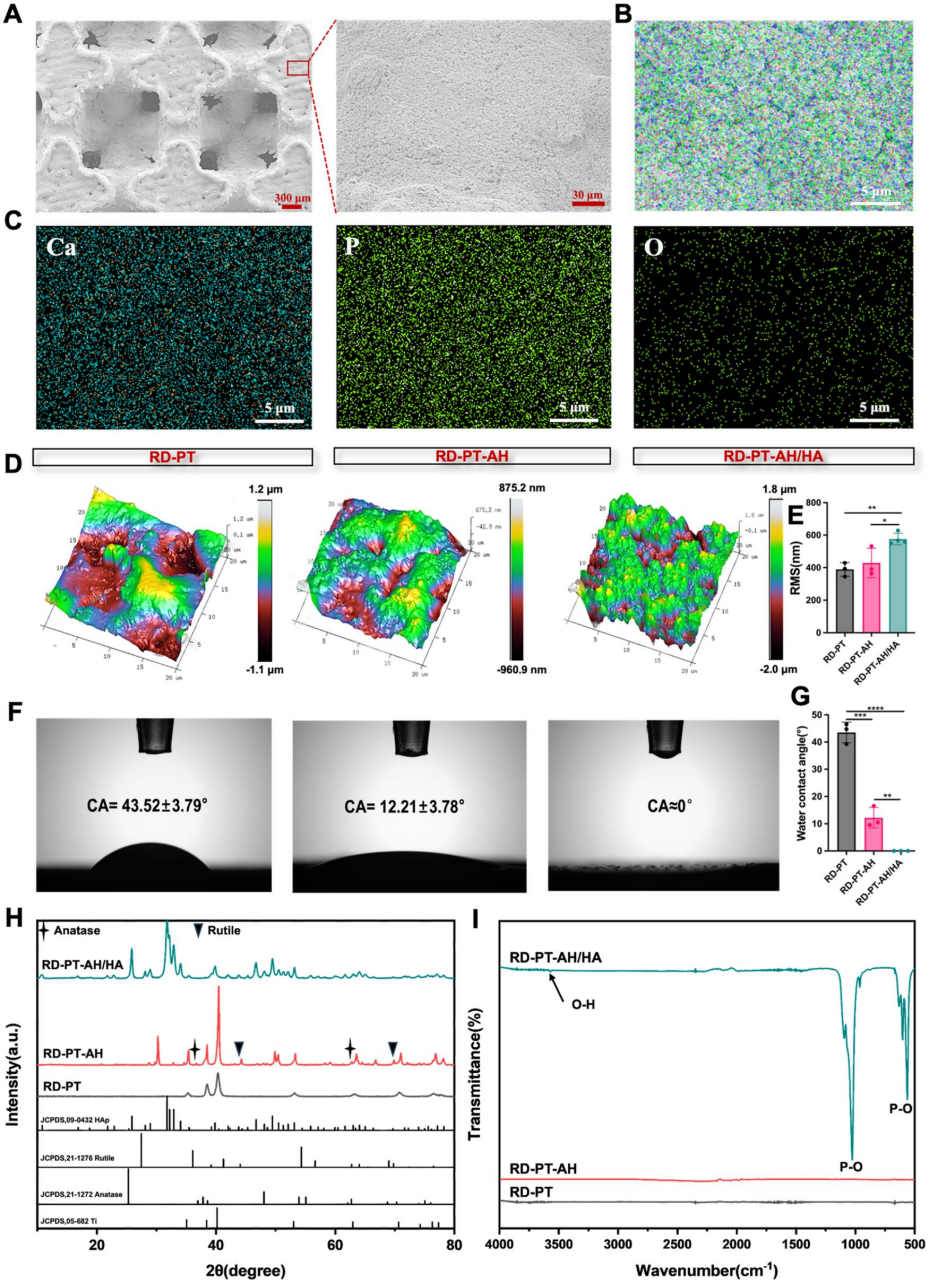
Surface characterization of samples at different modification stages. (**A**) SEM image of the RD structure scaffold after nHA modification. (**B**) EDS mapping and elemental distribution of the nHA coating. (**C**) Distribution of Ca, P and O elements within the nHA coating. (**D**) AFM surface roughness images and (**E**) the corresponding roughness values. (**F**) Morphology of water droplets on the sample surfaces and (**G**) the corresponding water contact angles. (**H**) X-ray diffraction patterns and (**I**) Fourier-transform infrared spectra. (*P < 0.05, **P < 0.01, ***P < 0.001, ****P < 0.0001).

Higher-magnification scanning electron microscopy (SEM) images (Figure 3A) revealed a relatively flat and smooth surface on the RD-PT scaffold. Following alkali-heat treatment, the surface roughness of RD-PT-AH increased and a microporous network structure formed due to surface etching in the strong alkaline environment. Subsequent vacuum perfusion of the nHA slurry onto the alkali-treated sample resulted in an nHA coating composed of numerous aggregated short-rod-like clusters, which exhibited a high specific surface area and distinct nanotopography. Cross-sectional SEM images (Figure 3B) clearly showed the coating-substrate interface for the differently modified samples. While the RD-PT surface remained smooth, a distinct microporous network layer with a thickness of approximately 1 µm was observed on RD-PT-AH. EDS line scanning (Figure 3C) confirmed the presence of an oxide layer on the RD-PT-AH surface, consistent with the SEM observations. The cross-section of RD-PT-AH/HA comprised three distinct layers: a top nHA coating, an intermediate alkali-heat-treated oxide layer and the underlying titanium alloy substrate. The average thickness of the nHA coating was measured to be 29.6 ± 4.79 µm (Figure 3D and Supplementary Figure S1). SEM results demonstrated the uniformity and microstructure of the coating (Figure 4A and Supplementary Figure S2). The nHA coating on all three scaffolds was uniformly bonded, free of cracks and did not obstruct the interconnected pore structure within the scaffolds. EDS area scanning (Figure 4B and C) confirmed the homogeneous distribution of Ca and P elements on the sample surface. Quantitative analysis yielded a Ca/P atomic ratio of 1.63 (Supplementary Table S2), which is very close to the theoretical value of 1.67 for hydroxyapatite (HA).

The surface coating significantly altered the physical properties of the material. Atomic force microscopy (AFM) analysis (Figure 4D and E) revealed that the RD-PT surface was relatively smooth with a root-mean-square roughness (Rq) of 389.00 ± 42.14 nm. Alkali-heat treatment slightly increased the surface roughness of RD-PT-AH, raising the Rq value to 429.00 ± 90.22 nm, which is consistent with the SEM observations. Subsequent vacuum perfusion with nHA further increased the roughness of RD-PT-AH/HA to an Rq of 575.75 ± 35.69 nm, a value significantly higher than those of both the RD-PT and RD-PT-AH groups. The coating, characterized by its nanoscale topography and hydrophilic groups, not only enhanced the average roughness but also improved the surface hydrophilicity (Figure 4F and G). Notably, both RD-PT-AH and RD-PT-AH/HA exhibited superior hydrophilicity compared to RD-PT, with RD-PT-AH/HA demonstrating superhydrophilic properties.

X-ray diffraction (XRD) analysis was performed to determine the crystalline phases of the surface coating (Figure 4H). Compared with the RD-PT group, the diffraction pattern of RD-PT-AH showed characteristic peaks attributed to rutile (JCPDS No. 21-1276) and anatase (JCPDS No. 21-1272). The XRD pattern of the RD-PT-AH/HA group displayed diffraction peaks that perfectly matched the standard pattern for hydroxyapatite (JCPDS No. 09-0432), confirming that the coating consisted of well-crystallized hydroxyapatite.

Furthermore, no diffraction peaks from other calcium phosphate impurity phases were detected, demonstrating the phase purity of the coating. Fourier-transform infrared (FTIR) spectroscopy (Figure 4I) further supported the above conclusions at the molecular functional group level. The absorption spectra of the RD-PT and RD-PT-AH groups showed no significant differences. In the spectrum of the RD-PT-AH/HA sample, characteristic vibrational absorption bands for the PO_4_^3−^ group were clearly observed: the doublet at 562 cm^−1^ and 602 cm^−1^ originated from the bending vibration of O-P-O, the absorption peak at 961 cm^−1^ corresponded to the P-O stretching vibration and the strong, broad absorption bands at 1047 cm^−1^ and 1092 cm^−1^ were attributed to the asymmetric stretching vibration of P-O. These serve as definitive evidence for the presence of HA.

### Bonding strength and *in vitro* degradation stability of the coatings

**Figure 5:**
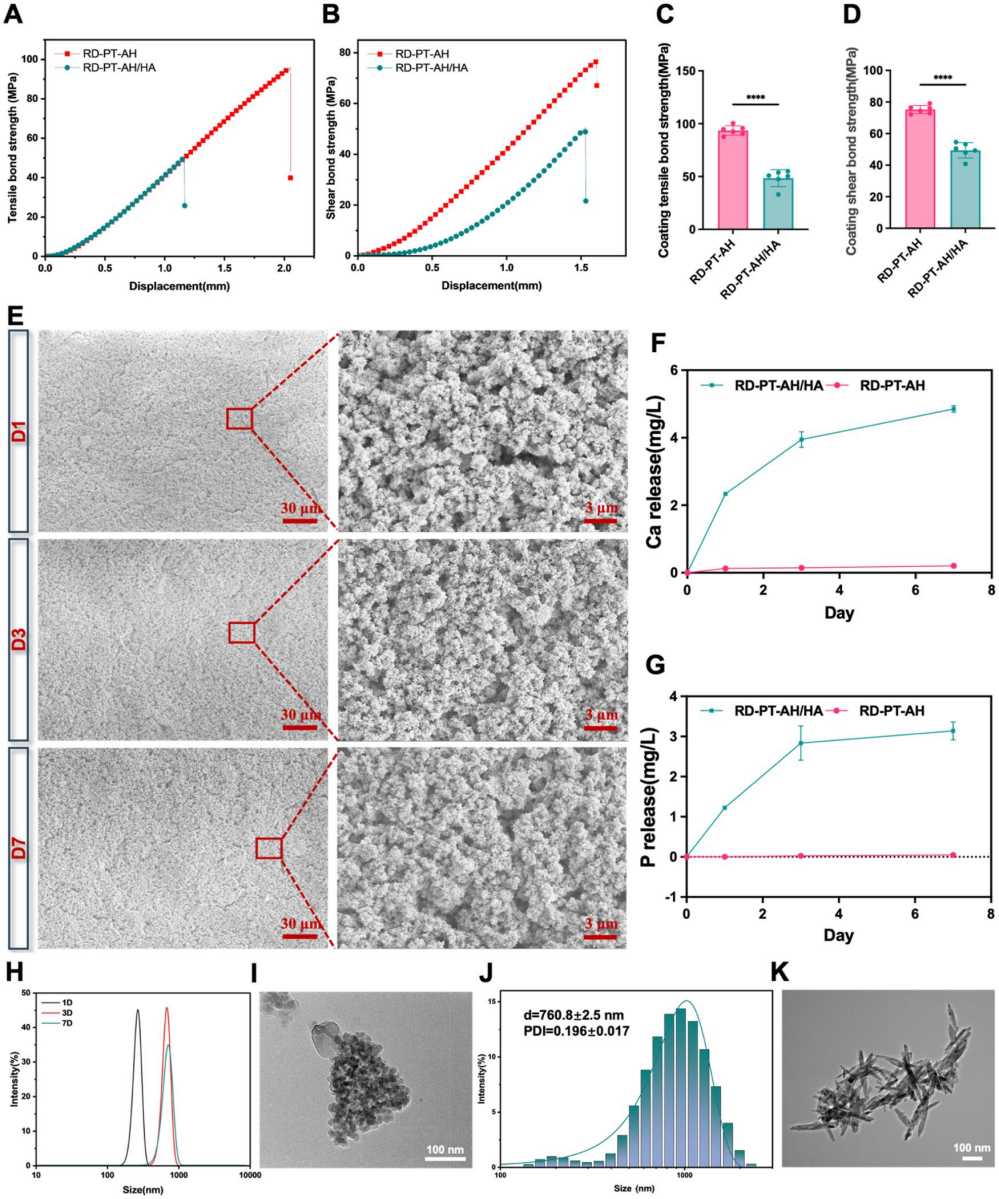
Mechanical properties and *in vitro* degradation behavior of the alkali-heat-treated oxide layer and the nHA coating. (**A**) Tensile bonding strength curves and (**C**) the corresponding values (*n* = 6). (**B**) Shear bonding strength curves and (**D**) the corresponding values (*n* = 6). (**E**) SEM morphology of the nHA coating after degradation and the release profiles of (**F**) calcium and (**G**) phosphorus ions. (**H**) Particle size distribution after nHA coating degradation and (**I**) TEM morphology of the degradation products after 7 days. (**J**) Particle size distribution and (**K**) TEM morphology of the original nHA slurry. (****P < 0.0001).

The bonding strength between the *in situ*-formed microporous network layer and the titanium alloy substrate, as well as between the nHA coating and the substrate, was quantitatively evaluated via tensile and shear adhesion tests (*n* = 6). The results (Figure 5A–D) revealed average tensile bonding strengths of 94.78 ± 5.14 MPa and 48.50 ± 7.31 MPa, respectively, and average shear bonding strengths of 75.20 ± 2.34 MPa and 49.40 ± 4.47 MPa, respectively. Given that the *in vitro* degradation stability of the nHA coating is crucial for its long-term biological function, degradation tests were conducted on the RD-PT-AH and RD-PT-AH/HA groups. As shown in Figure 5E, SEM images revealed the morphological evolution of the nHA coating surface during degradation. On Day 1, the coating surface was uniform, and the short-rod-like nHA particles exhibited sharp, distinct contours at high magnification. By Day 3, the surface remained uniform at low magnification, but the particle contours became blunted at higher magnification, indicating initial mild degradation. By Day 7, further mild degradation was observed compared to Day 3. The concentrations of Ca^2+^ and PO_4_^3-^ ions in the degradation medium were further measured. As shown in Figure 5F and G, throughout the testing period, the Ca^2+^ and PO_4_^3-^ concentrations for the RD-PT-AH group remained stable, indicating no ion release. In contrast, for the RD-PT-AH/HA group, the concentrations of Ca^2+^ and PO_4_^3-^ ions gradually increased over time, with the rate of increase slowing down progressively.

By Day 7, the changes in ion concentration leveled off, reaching a plateau. Elemental analysis revealed no evidence of coating disintegration or failure. The ion release profiles aligned with the observed microstructural changes, collectively suggesting that the nHA coating underwent uniform and gradual degradation. After 7 days of immersion, the particle size distribution of the released nHA particles was comparable to that on Day 3, with an average size of approximately 712.0 nm (Figure 5H). TEM analysis showed that these particles maintained their crystalline morphology but exhibited reduced dimensions and more rounded edges compared to the original nHA slurry (Figure 5I). The original nHA particles had a size distribution of approximately 760.8 nm (Figure 5J) and displayed a nanoneedle rod-shaped morphology (Figure 5K). These nano-sized primary particles constitute the micro-nano hierarchical structure of the coating. Overall, these results demonstrate that the nHA coating degrades in a controlled and slow manner within a physiological environment, primarily via surface dissolution rather than structural disintegration.

### *In vitro* osteogenic activity of 3D-printing titanium alloy scaffolds

**Figure 6:**
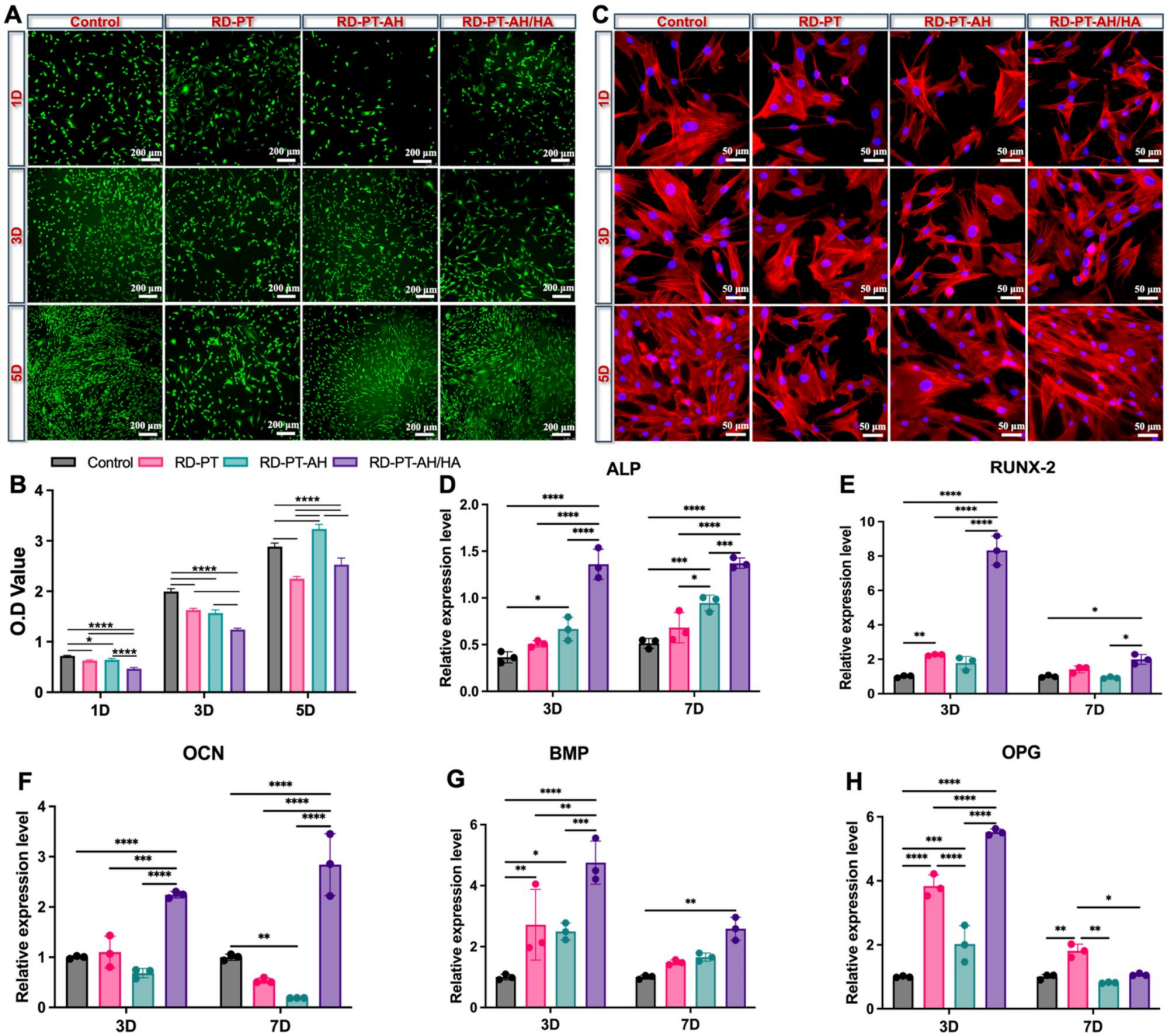
*In vitro* osteogenic activity of the porous titanium alloy scaffolds. (**A**) CLSM images of cells stained with FDA and PI. (**B**) Cell viability of BMSCs after co-culture with different samples. (**C**) CLSM images of the cytoskeleton and nuclei stained with TRITC-Phalloidin and DAPI. (**D–H**) Expression of osteogenic genes in BMSCs after co-culture with different samples, analyzed by qRT-PCR. (*P < 0.05, **P < 0.01, ***P < 0.001, ****P < 0.0001).

Representative fluorescence microscopy images of bone marrow mesenchymal stem cells (BMSCs) stained with FDA-PI after 1, 3 and 5 days of culture on the various sample surfaces are shown in Figure 6A. Cells exhibited robust growth across all groups with minimal cell death. BMSC density increased progressively over time, and cell viability remained consistently high, which aligns with the quantitative CCK-8 results (Figure 6B). On Day 1 and 3, cell density and viability were slightly higher in the RD-PT and RD-PT-AH groups compared to the RD-PT-AH/HA group. By Day 5, however, both the RD-PT-AH and RD-PT-AH/HA groups supported significantly greater cell density and viability than the RD-PT group, indicating enhanced cytocompatibility after surface modification. Cell-spreading morphology was further evaluated via cytoskeleton and nuclear staining (Figure 6C). Cells on all modified surfaces showed well-extended polygonal or spindle-shaped morphologies with abundant, organized stress fibers and numerous peripheral lamellipodia and filopodia. Nuclei maintained a regular elliptical shape. Compared to the RD-PT and RD-PT-AH groups, cells seeded on the RD-PT-AH/HA surface exhibited higher spreading density and more developed stress fiber formation.

The influence of surface modification on the expression of key osteogenic genes in BMSCs was further investigated (Figure 6D–H, primer sequences are listed in Supplementary Table S3). Expression of the early marker alkaline phosphatase (ALP) was upregulated in both the RD-PT-AH and RD-PT-AH/HA groups, with a more pronounced effect in the latter, while the RD-PT group showed no significant difference from the blank control. Compared to the control, RUNX-2 expression was elevated 8.3-fold in the RD-PT-AH/HA group on Day 3, indicating an early activation of the osteogenic program. Although its expression declined by Day 7, it remained significantly higher than in both the control and the RD-PT-AH group. A similar trend was observed for BMP, which was upregulated 4.8-fold in the RD-PT-AH/HA group on Day 3. Differences in mid-to-late-stage markers became more distinct with prolonged culture. Osteocalcin (OCN) expression was significantly upregulated in the RD-PT-AH/HA group at both Day 3 and Day 7. Notably, osteoprotegerin (OPG) was initially induced 5.5-fold in the RD-PT-AH/HA group, suggesting that the modified surface may favor osteogenesis by upregulating OPG to suppress osteoclast activity. Collectively, these results demonstrate that the nHA coating effectively promotes the osteogenic differentiation of BMSCs.

### *In vivo* osteoinductivity of 3D-printing titanium alloy scaffolds

**Figure 7:**
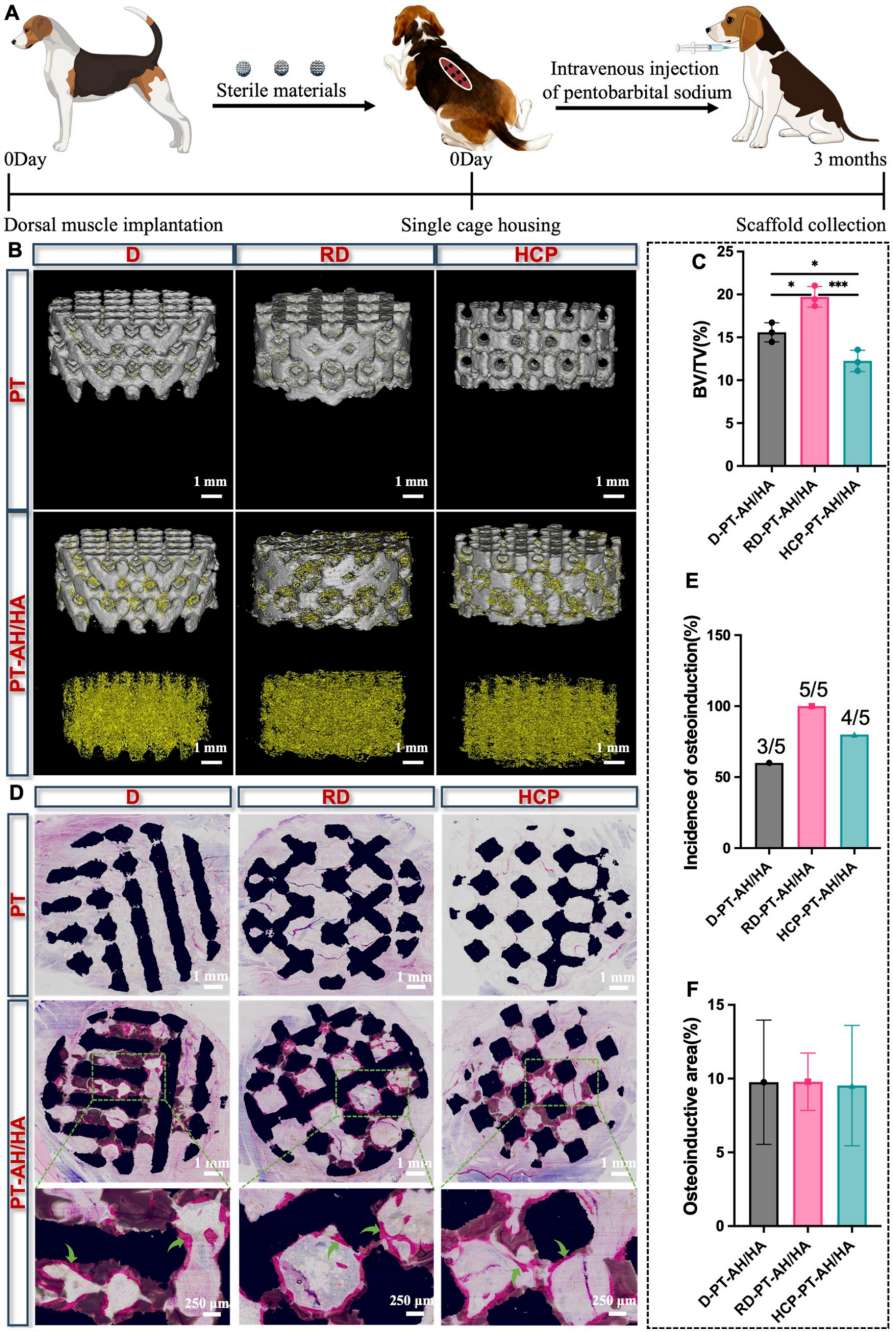
*In vivo* osteoinductivity of the porous titanium alloy scaffolds. (**A**) Schematic diagram of the *in vivo* animal experiment. (**B**) Representative micro-CT images of the explanted samples after 3 months of implantation. (**C**) Percentage of new bone volume/total pore volume (BV/TV) calculated from the micro-CT data. (**D**) Microscopy images of hard-tissue sections stained with methylene blue-basic fuchsin. (**E**) Osteogenic induction success rate (*n* = 5). (**F**) Quantitative analysis of the osteogenic area based on staining results. (*P < 0.05, ***P < 0.001).

To assess the osteoinductive capacity of nHA-coated porous titanium alloy scaffolds, as-printed (D-PT, RD-PT, HCP-PT) and nHA-coated (D-PT-AH/HA, RD-PT-AH/HA, HCP-PT-AH/HA) scaffolds were implanted ectopically into the dorsal muscle of beagle dogs as control and experimental groups, respectively (Figure 7A). *Ex vivo* micro-CT imaging (Figure 7B) showed scant mineralized signals in the control groups, indicating negligible bone formation. In contrast, scaffolds subjected to alkali-heat treatment and nHA-coating exhibited clear, high-density mineralization within their pores, confirming uniform new bone deposition along the inner surfaces. Quantitative analysis revealed that the RD-PT-AH/HA group achieved the highest bone volume fraction (BV/TV) at 19.71%, followed by D-PT-AH/HA (15.59%) and HCP-PT-AH/HA (12.25%) (Figure 7C).

To further validate the *ex vivo* micro‑CT findings, histological analysis was performed on tissue sections stained with methylene blue‑basic fuchsin. As shown in Figure 7D, the control scaffolds exhibited minimal osteogenesis. Their pores were predominantly filled with fibrous connective tissue, showing no signs of osteoblast activity or bone matrix deposition. The scaffold‑tissue interface mainly consisted of collagen fibers with sporadic capillaries, lacking a functional vascular network to support bone formation. In contrast, all three nHA‑coated scaffold types in the experimental groups displayed substantial new bone formation (indicated by arrows), with active osteoblasts and clear deposition of bone matrix. The osteoinduction success rate was statistically evaluated, and the area of newly formed bone was quantified using Image‑Pro Plus software. As shown in Figure 7E, the RD structure achieved the highest osteoinduction rate of 100% (*n* = 5), indicating excellent osteoinductive potential after nHA-surface modification. The osteogenic area fraction was similar across all three coating groups, each approaching 10% (Figure 7F).

### *In vivo* osseointegration performance of 3D-printing titanium alloy scaffolds

**Figure 8:**
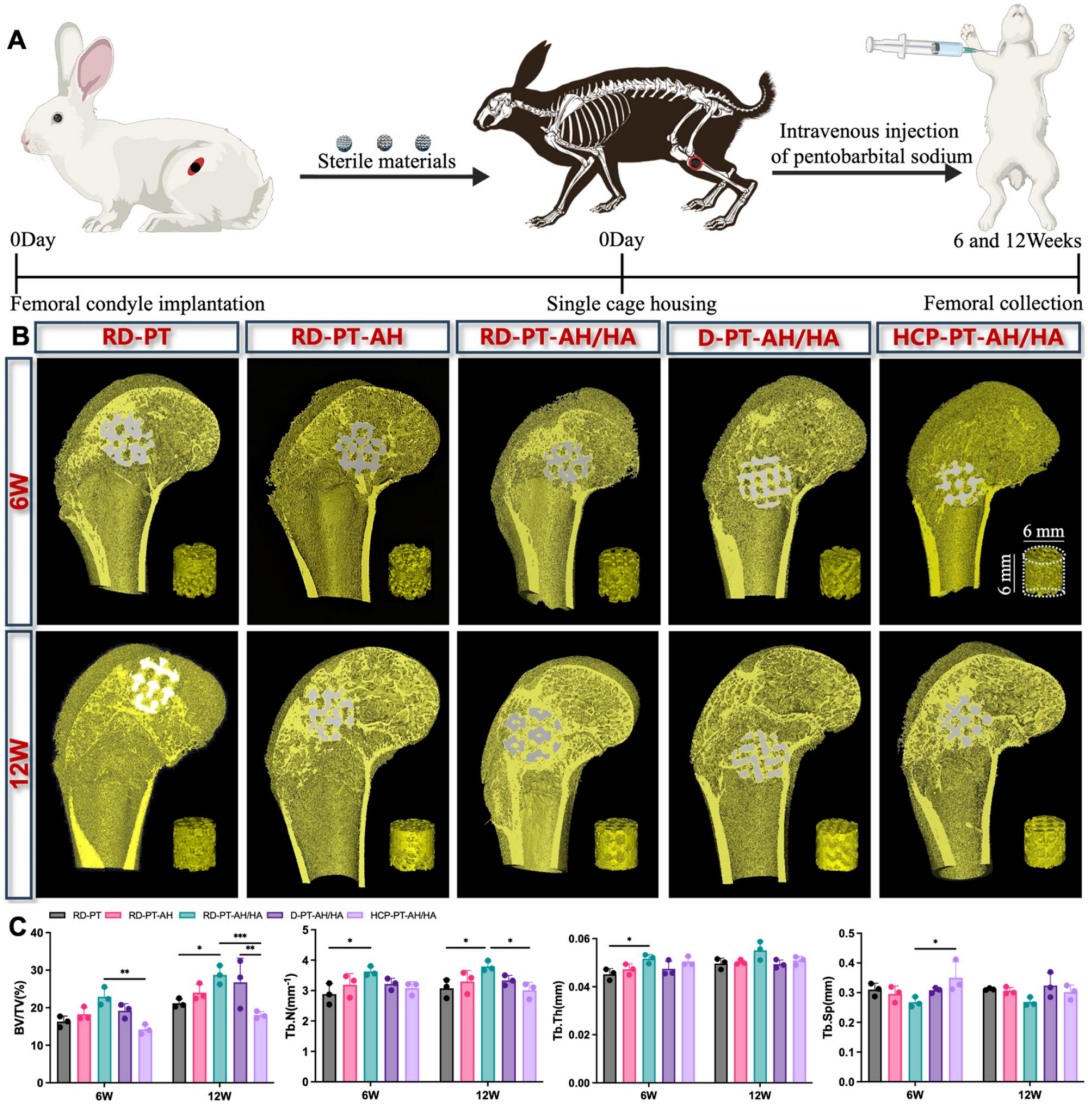
*In vivo* osseointegration performance of the porous titanium alloy scaffolds. (**A**) Schematic diagram of the *in vivo* animal experiment. (**B**) Reconstructed micro-CT images. (**C**) Quantitative osseointegration parameters derived from micro-CT data, including bone volume fraction (BV/TV), trabecular number (Tb.N), trabecular thickness (Tb.Th) and trabecular separation (Tb.Sp). (*P < 0.05, **P < 0.01, ***P < 0.001).

To systematically investigate the synergistic effect between pore structure and surface chemistry with minimal animal experimentation, we chose the best-performing RD-structured porous scaffolds in their differently modified forms (RD-PT, RD-PT-AH, RD-PT-AH/HA) along with two other structurally distinct nHA-coated scaffolds (D-PT-AH/HA and HCP-PT-AH/HA) for implantation into critical-sized femoral condyle defects in New Zealand white rabbits to evaluate their *in vivo* bone regeneration and osseointegration capabilities. Figure 8A depicts the experimental workflow and timeline for the femoral condyle defect model in New Zealand white rabbits. Three-dimensional reconstructed micro-CT images at 6 and 12 weeks postoperation (Figure 8B) clearly delineated the spatiotemporal changes and differences in new bone ingrowth among the groups. Reconstructed cross-sectional views distinctly showed that new bone formation was more pronounced at 12 weeks than at 6 weeks in all groups. Notably, the RD-PT-AH/HA group exhibited excellent integration between newly formed bone and the implant, which was markedly superior to that in the other experimental groups. Quantitative analysis of the reconstructed data (Figure 8C) revealed significant differences in bone ingrowth among scaffolds with different architectures, even under identical surface treatment conditions (all with nHA coating). At both 6 and 12 weeks, the RD-PT-AH/HA group showed the highest bone volume fraction (BV/TV), with values of 22.9 ± 2.4% and 28.7 ± 2.5%, respectively. These values were significantly higher than those of the D-PT-AH/HA and HCP-PT-AH/HA groups. For the RD structure, surface modification significantly improved osteogenic outcomes, and the BV/TV values followed a clear hierarchy: RD-PT-AH/HA > RD-PT-AH > RD-PT. Furthermore, analysis of trabecular parameters consistently revealed that the RD-PT-AH/HA group exhibited superior osteogenic performance.

**Figure 9:**
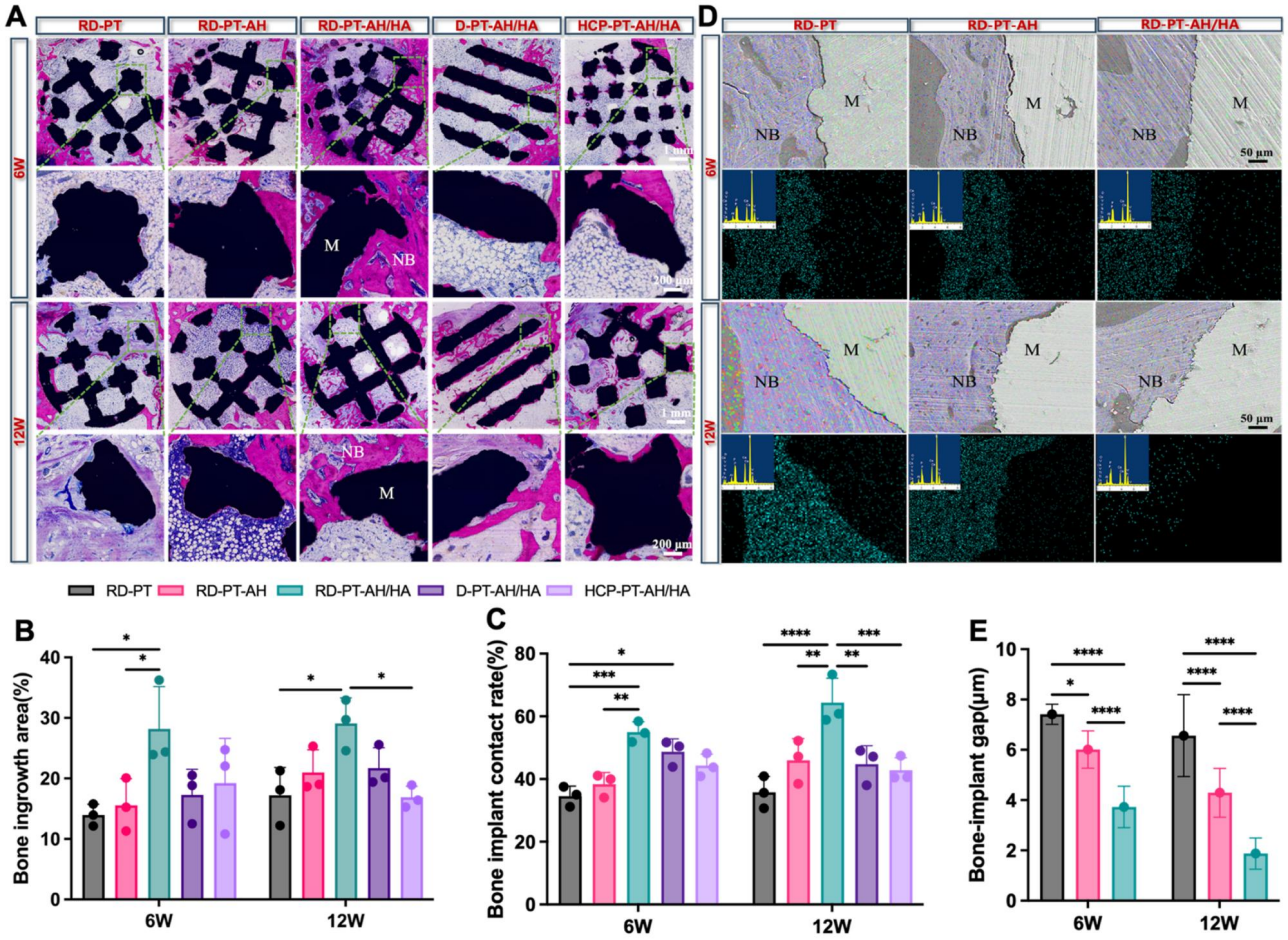
Staining of bone tissue sections and evaluation of the gap between new bone tissue and the implant. (**A**) Microscopic images of tissue sections stained with methylene blue–basic fuchsin. (**B**) Percentage of osteogenic area calculated based on stained sections. (**C**) Bone-implant contact (BIC) rate calculated from the stained sections. (**D**) Scanning electron microscopy (SEM) image of a tissue section and the corresponding EDS elemental map for calcium. (**E**) Gap between new bone tissue and the implant. (*P < 0.05, **P < 0.01, ***P < 0.001, ****P < 0.0001).

The methylene blue–basic fuchsin staining results (Figure 9A) provided direct histological evidence of the osseointegration outcomes. In the RD-PT and RD-PT-AH groups, newly formed bone (stained purplish-red) was largely confined to the host bone margins with minimal ingrowth into the scaffold interior. In contrast, the RD-PT-AH/HA, D-PT-AH/HA and HCP-PT-AH/HA groups all demonstrated extensive new bone tissue ingrowth deep into the pores, establishing direct contact with the implant surface. The RD-PT-AH/HA group, in particular, exhibited a larger area of more mature new bone, which displayed a typical lamellar structure and formed a mechanically interlocked bone-bridging structure with the scaffold.

Furthermore, quantitative analysis (Figure 9B and C) revealed that the RD-PT-AH/HA group had the highest bone ingrowth area at both 6 and 12 weeks postoperation, with values of 28.2 ± 7.0% and 29.1 ± 4.2%, respectively. The bone-implant contact (BIC) rate followed a similar trend, reaching 55.0 ± 3.3% and 64.4 ± 7.8% in the RD-PT-AH/HA group at 6 and 12 weeks, respectively, which indicates rapid and high-quality osseointegration. The bone-implant interface was further examined using SEM and EDS mapping (Figure 9D and E). Both EDS maps and statistical analysis of the interfacial gap showed a progressive tightening of the bone-implant contact with an increased degree of modification, characterized by a significant gradient reduction in the gap width. The RD-PT-AH/HA group displayed the most intimate contact with the new bone, showing a virtually gap-free interface. In the EDS maps, purple regions indicate newly formed bone, light grey areas correspond to the implant scaffold and the blue–green overlay indicates the calcium distribution within the new bone, providing visual confirmation of the superior osseointegration in the RD-PT-AH/HA group. These findings confirm that the nHA-coating possesses superior bioactivity compared to alkali-heat treatment alone or the pristine titanium surface.

## Discussion

For a long time, titanium and its alloys have been the preferred materials for bone implants due to their excellent mechanical properties and biocompatibility. However, issues such as excessively high modulus and low bioactivity still persist, significantly affecting the long-term clinical stability and repair efficacy of titanium-based implants [38, 39]. Although integrating topological optimization with surface coating strategies can effectively lower the elastic modulus and enhance the bioactivity of titanium-based implants, it remains challenging to achieve a balance among topological structure, coating uniformity and adhesion strength. In this study, based on the CFD simulations and 3D printing technology, we developed a simple gradient liquid-phase impregnation method to construct a uniform, crack-free nHA coating with high adhesion strength on porous titanium alloy, endowing it with excellent osteoinductive and osseointegrative capabilities.

The topological structure is a key factor affecting the mechanical properties of scaffolds, with an appropriate design ensuring high internal connectivity and structural stability, as well as excellent strength [40–42]. Benefiting from smoother pore transitions and uniform nodal designs, the RD lattice scaffold exhibits structural and mechanical characteristics that are more closely matched to natural bone compared to D lattice and HCP lattice scaffolds (Figure 2), effectively reducing the risk of stress shielding. More importantly, the scaffold provides a favorable hydrodynamic microenvironment for cell differentiation and tissue growth, characterized by high permeability for efficient transport of nutrients, metabolites and signaling molecules. In addition, regions with a WSS below 10 mPa can significantly upregulate F-actin expression, promote the formation of stress fibers and focal adhesions in the cytoskeleton and consequently activate relevant signaling pathways to regulate cytoskeletal contractility and tension. This subsequently activates downstream mechanosensitive factors, thereby effectively driving osteogenic differentiation of the cells [33, 43, 44].

The bonding strength between the bioactive coating and the implant surface is crucial for ensuring the long-term stability of the implant and its clinical repair effectiveness [45]. Among the various coating methods currently available, the coating constructed by high-temperature plasma spraying exhibits the highest bonding strength and achieves the best repair outcomes in clinical applications [46]. However, it is difficult to apply to porous implants. In this study, we first pretreated the surface of the porous titanium alloy scaffold through alkali-heat treatment to form a nanoscale microporous oxide layer (Figure 3). This layered structure not only eliminates residual stresses and partially mitigates micro-stress concentrations introduced during the 3D printing process but also provides abundant sites for the subsequent deposition of nHA. After gradient vacuum infusion, the nHA penetrates deep into the pores of the scaffold, filling the micropores in the pore walls and forming a well-interlocked structure. Furthermore, low-temperature heat treatment is applied to the scaffold after nHA deposition. This process removes the organic binder from the slurry while promoting the transformation of some amorphous calcium phosphate into crystalline HA, further enhancing the interfacial bonding between the nHA coating and the substrate. As shown in Figure 5, the bonding strength of the nHA coating prepared in this study is comparable to that of HA coatings produced by plasma spraying methods reported in other studies, meeting the clinical requirements for the bonding strength of nHA coatings on implants [47, 48]. Generally, during processes such as high-temperature thermal plasma spraying and electrochemical treatment for coating deposition, the scaffold substrate may sustain certain damage due to excessive thermal stress or electrochemical corrosion [49–51]. In contrast, the nanocoatings prepared in this study not only avoid such issues but also enhance the mechanical strength of the titanium alloy scaffold substrate, providing a reinforcing effect.

Due to the unique nanoscale effects and hydrophilic functional groups of nHA, the coated scaffolds exhibit significantly improved surface roughness and hydrophilicity (Figure 4D–G). Additionally, these scaffolds can sustainably release Ca^2+^ and PO_4_^3-^ ions (Figure 5E–G), thereby creating a beneficial physiological microenvironment for subsequent cell adhesion and growth. Compared to the unmodified RD-PT, the alkali-heat-treated (RD-PT-AH) and coated (RD-PT-AH/HA) scaffolds exhibited larger cell-spreading areas (Figure 6A and C), indicating that their roughened hydrophilic nanostructures provided more anchoring sites for cells, promoting adhesion and cytoskeleton assembly. Subsequently, the nHA coating began to continuously release Ca^2+^ and PO_4_^3-^ ions, which interact with specific receptors such as the calcium-sensing receptor (CaSR) on the cell membrane to activate the downstream MAPK/ERK and Wnt/β-catenin osteogenic signaling pathways. This activation facilitates the initiation of the osteogenic program at the transcriptional level, resulting in a significant upregulation of osteogenic-related gene expression [52–55]. Notably, the expression levels of RUNX-2, BMP and OPG in the RD-PT-AH/HA group were lower on Day 7 than on Day 3, which follows the normal temporal pattern of osteogenic differentiation. As an early core transcription factor, RUNX-2 peaks during the initial differentiation stage and then naturally downregulates during matrix maturation and mineralization [56, 57]. Similarly, BMP acts as an upstream signal that declines after the downstream osteogenic program is activated [58]. Although OPG is a late-stage regulator, its high expression on Day 3 may indicate an early response of osteogenic cells to the microenvironment, while the decrease on Day 7 reflects the functional transition as cells exit proliferation and enter matrix maturation [59].

This enhanced regulation of cell differentiation further allows the coated scaffold to promote protein adsorption and the recruitment of surrounding stem cells following intramuscular implantation [60, 61], which then proceed to osteogenic differentiation under the mediation of relevant signaling pathways. Concurrently, the release and redeposition of Ca^2+^ and PO_4_^3-^ ions facilitate the mineralization and formation of bone matrix, ultimately leading to the generation of bone tissue [62, 63]. However, it should be noted that although all three types of lattice scaffolds modified with nHA coatings exhibited signs of bone formation after 12 weeks of heterotopic implantation, the RD lattice scaffold demonstrated the highest incidence of osteoinduction (5/5) and the highest bone formation volume fraction (19.71%), indicating stronger osteoinductive capabilities (Figure 7). Under identical modification conditions, this difference in osteoinductive ability can be attributed to the distinct hydrodynamic microenvironment resulting from topological structures. Compared to D lattice and HCP lattice scaffolds, the RD lattice scaffold features deeper fluid permeability and lower interfacial shear stress, which enhance the recruitment of cells into the scaffold and ensure their adhesion and growth within the pore spaces. This favorable hydrodynamic microenvironment, combined with abundant Ca and P sources, promotes the rapid recruitment of stem cells from the physiological environment into the scaffold and accelerates their differentiation into bone tissue in the modified porous titanium alloy scaffold.

After implantation into critical bone defects, the nHA-coated modified scaffolds with osteoinductive capabilities demonstrated significantly enhanced bone ingrowth and interfacial osseointegration compared to unmodified titanium alloy scaffolds. As can be clearly observed in Figure 9, the modified scaffolds integrated more closely with the host bone, with abundant mature bone tissue occupying the internal spaces of the scaffolds. Moreover, similar to the trend observed in osteoinductive ability, the RD lattice scaffold exhibited significantly superior bone regeneration and interfacial osseointegration compared to the D lattice and HCP lattice scaffolds. This is related not only to the extent of its osteoinductive potential but also likely to how closely matched the scaffold and bone tissue are in terms of structure and mechanics. For example, the RD scaffold possesses a pore size of 500–700 μm and a porosity of 60% to 70%, a range widely recognized as optimal for bone ingrowth [64–66]. Furthermore, its unique nodal and strut design likely creates a physical space that is more conducive to vascular ingrowth, cell migration and bone matrix deposition. The RD lattice scaffold, with its superior structural and mechanical matching, allows for more effective interfacial stress transmission, guides the migration of cells from surrounding tissues into the defect site and enables endogenous bone regeneration. As noted in the studies by Garner, Chen and others, implants with suitable topological structures can improve interfacial load transfer through being more closely matched with host bone tissue after implantation; significantly reduce the incidence of interfacial fractures caused by mechanical loading; and enhance postoperative bone remodeling [67, 68].

## Conclusion

In this study, an RD porous titanium alloy scaffold with an optimized fluidic microenvironment was successfully developed by integrating CFD simulations with SLM 3D printing technology. This structure demonstrated excellent permeability and a uniform shear stress distribution. Furthermore, a uniform, crack-free nano-hydroxyapatite (nHA) coating with suitable bonding strength was constructed on the scaffold surface via alkali-heat treatment and gradient vacuum perfusion techniques. *In vitro* experiments demonstrated that the nHA coating significantly promoted the adhesion, proliferation and osteogenic differentiation of BMSCs by enhancing surface hydrophilicity and providing nanotopography. In an ectopic implantation model within the dorsal muscle of beagle dogs, this scaffold achieved a 100% osteoinduction rate. Moreover, it exhibited superior osseointegration performance in a rabbit femoral condyle critical-sized defect model. This work demonstrates the synergistic effect of topological optimization and surface modification, providing a novel strategy and methodology for developing high-performance orthopedic implants.

## Supplementary Material

rbag090_Supplementary_Data
