# Supplementary material for: Topological optimization synergized with a high-activity nano-hydroxyapatite coating to enhance bone regeneration in a porous titanium alloy scaffold
Source: Regen Biomater. 2026 May 7;13:rbag090. doi: 10.1093/rb/rbag090 (PMC13289606; doi:10.1093/rb/rbag090)
Supplement: rbag090_Supplementary_Data [file rbag090_supplementary_data.zip › Supplementary material.docx]

**Topological optimization synergized with a high-activity nano-hydroxyapatite coating to enhance bone regeneration in porous titanium alloy scaffold**

*Peng Zhao ^1,2^, Yushuan Jia ^1,2^, Jingming Li^1,2^, Fengyi You ^3^, Kai Zhang^1,2^, Bo Yuan ^1,2,*^, Xiangdong Zhu ^1,2,*^*

^1^ National Engineering Research Center for Biomaterials, Sichuan University, Chengdu, 610064, China

^2^ College of Biomedical Engineering, Sichuan University, Chengdu, 610064, China

^3^ Changzhou Jishuo Medical Device Co., Ltd., Changzhou, 213146, China

*Corresponding authors: Bo Yuan (scuyb@scu.edu.cn)

Xiangdong Zhu (zhu_xd1973@scu.edu.cn)


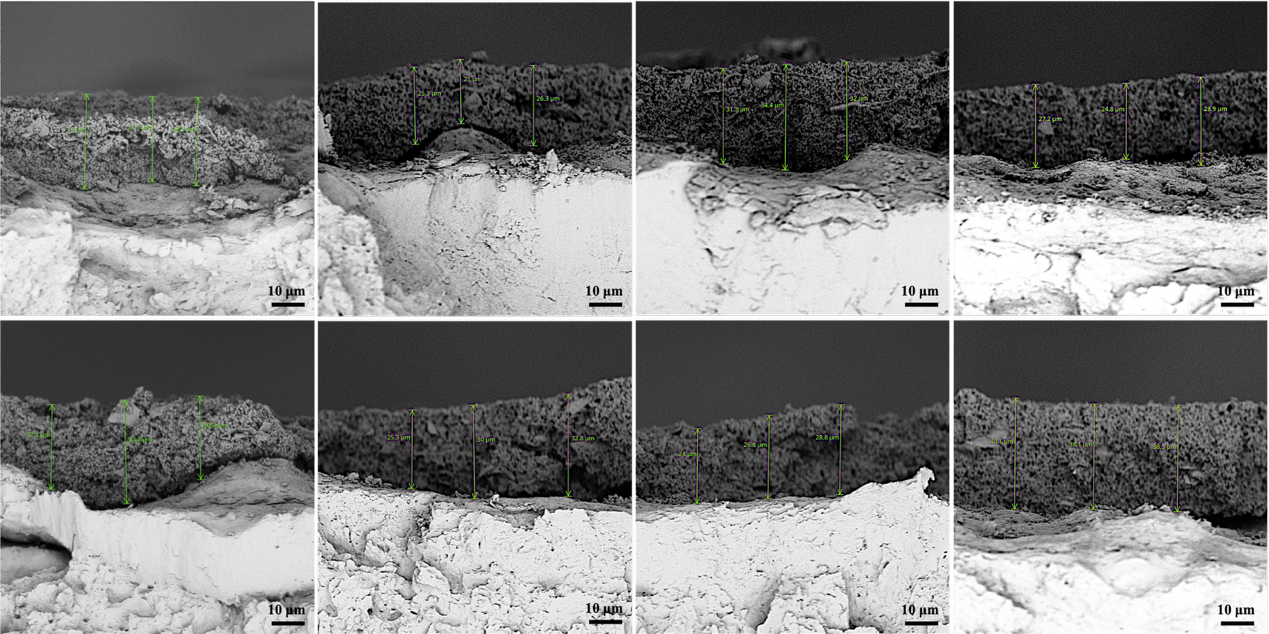


**Figure S1.** Thickness analysis of the nHA coating.

**
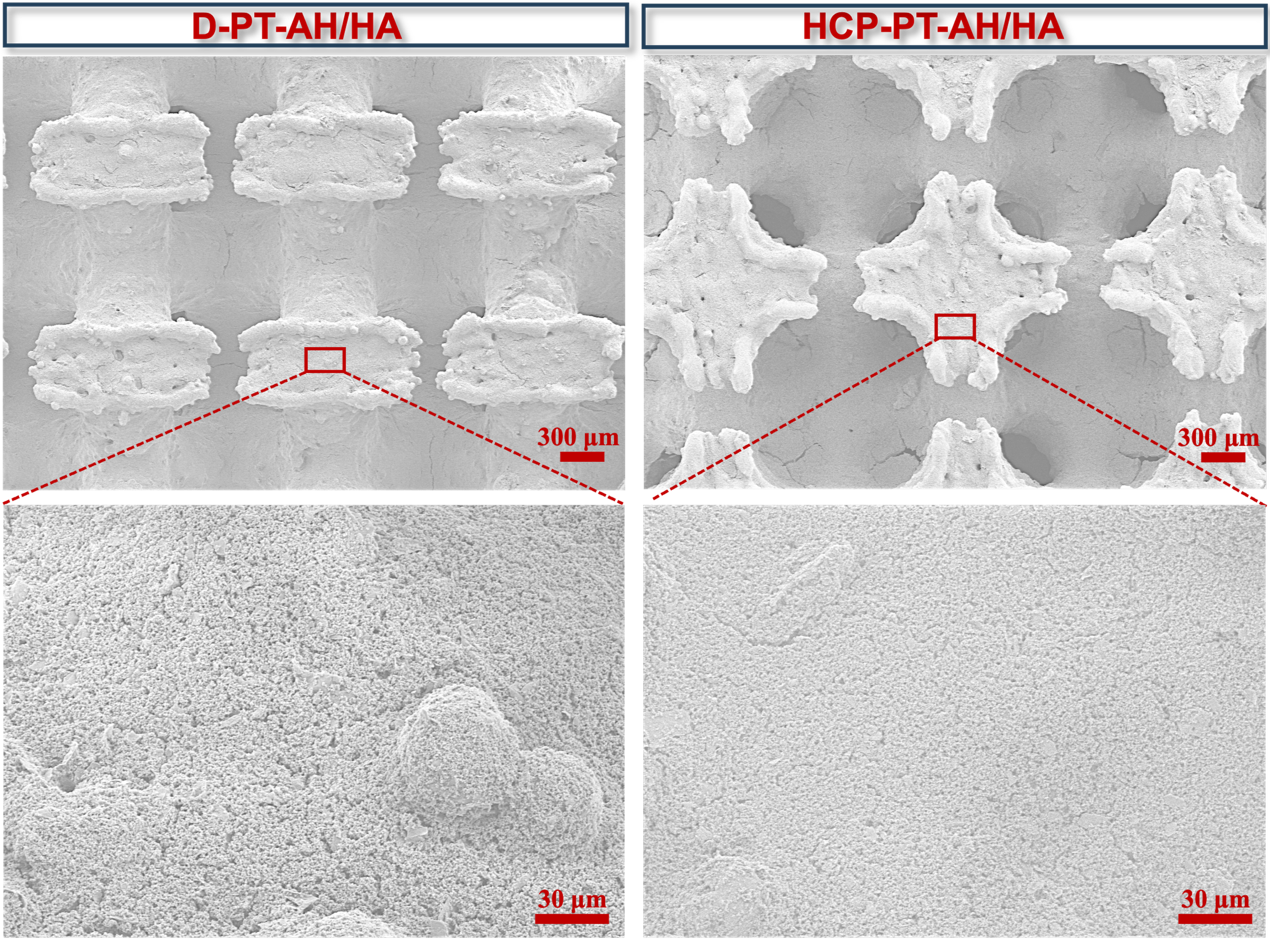
**

**Figure S2. SEM images of scaffolds with diamond (D) and hexagonal close-packed (HCP) structures after nHA modification.**

**Table S1. Composition indexes and compressive properties of porous scaffolds with three structures**

| **Structure of scaffolds** | **Unit Cell Size**  **(mm)** | **Strut Diameter**  **(mm)** | **Porosity**  **(%)** | **Diameter of the**  **Insphere**  **(μm)** | **Equivalent Diameter of Through-Pores** | **Compressive Strength**  **(MPa)** |
| --- | --- | --- | --- | --- | --- | --- |
| Diamond | 2.0 | 0.6 | 66.63 | 653 | 814.2 | 112.53±2.74 |
| Rhombic Dodecahedron | 2.9 | 0.6 | 66.43 | 1118 | 583.9 | 132.26±3.18 |
| Hexagonal Close-Packed | 0.88 | 0.3 | 75.07 | 550 | 550.0 | 105.70±2.53 |

**Table S2. Elemental composition and atomic ratios of the nHA coating obtained by EDS area scanning.**

| **Element Symbol** | **Element Name** | **Atomic Conc.（%）** |
| --- | --- | --- |
| O | Oxygen | 65.15 |
| Ca | Calcium | 21.60 |
| P | Phosphorus | 13.25 |

**Table S3. qRT-PCR Primer sequences for osteogenic-related genes**

| Target | Forward primer | Reverse primer |
| --- | --- | --- |
| ALP | CATCGCCTATCAGCTAATGCACA | ATGAGGTCCAGGCCATCCAG |
| RUNX-2 | TGGCCGGGAATGATGAGAAC | TTGAACCTGGCCACTTGGTT |
| BMP | TGACTGGATCGTGGCACCTC | CAGAGTCTGCACTATGGCATGGTTA |
| OCN | TCTGAGTCTGACAAAGCCTTCAT | AAGTCCATTGTTGAGGTAGCG |
| OPG | AACCGCACCCACAACCGA | CACCTGAGAAGAACCCATCCG |
| GADPH | GGCACAGTCAAGGCTGAGAATG | ATGGTGGTGAAGACGCCAGTA |
